# A Novel Detection Method of Breast Cancer through a Simple Panel of Biomarkers

**DOI:** 10.3390/ijms231911983

**Published:** 2022-10-09

**Authors:** Alinne T. F. Silva, Cláudia M. Rodrigues, Izabella C. C. Ferreira, Letícia L. D. Santos, Donizeti W. Santos, Thaise G. Araújo, Paula P. L. Canto, Carlos E. Paiva, Luiz R. Goulart, Yara C. P. Maia

**Affiliations:** 1 Molecular Biology and Nutrition Research Group, School of Medicine, Graduate Program in Health Science, Av. Amazonas sn, Block 2E, 2º Floor, Room 210, Campus Umuarama, Uberlandia 38405-320, Minas Gerais, Brazil; 2 Laboratory of Nanobiotechnology Prof. Dr. Luiz Ricardo Goulart Filho, Institute of Biotechnology, Federal University of Uberlandia, Av. Amazonas sn, Block 2E, 2º Floor, Room 248, Campus Umuarama, Uberlandia 38405-302, Minas Gerais, Brazil; 3 Obstetric Division, University Hospital, Federal University of Uberlandia, Av. Pará, 1720, Block 2H, Campus Umuarama, Uberlandia 38405-320, Minas Gerais, Brazil; 4 Department of Clinical Oncology, Clinics Hospital, Federal University of Uberlandia, Av. Pará, 1720, Oncology Sector, Room 9, Campus Umuarama, Uberlandia 38405-320, Minas Gerais, Brazil; 5 Department of Clinical Oncology, Graduate Program in Oncology, Palliative Care and Quality of Life Research Group (GPQual), Barretos Cancer Hospital, R. Antenor Duarte Vilela, 1331, Doutor Paulo Prata, Barretos 14784-400, Sao Paulo, Brazil

**Keywords:** breast cancer, circulating tumor cells, epithelial–mesenchymal transition, diagnosis, treatment monitoring

## Abstract

Circulating tumor cells (CTCs) have been identified as responsible for the spread of tumors to other organs of the body. In this sense, the development of sensitive and specific assays for their detection is important to reduce the number of deaths due to metastases. Here, we assessed whether the detection of CTCs in peripheral blood can serve in the construction of a panel of diagnosis and monitoring treatments of breast cancer (BC), focusing on the expression of markers of epithelial–mesenchymal transition. Through analyzing the blood from women without breast alterations (control), women with benign alterations, women with breast cancer without chemotherapy, and women with breast cancer with chemotherapy, we identified the best markers by transcriptional levels and determined three profiles of CTCs (mesenchymal, intermediate, and epithelial) by flow cytometry which, combined, can be used for diagnosis and therapy monitoring with sensitivity and specificity between 80% and 100%. Therefore, we have developed a method for detecting breast cancer based on the analysis of CTC profiles by epithelial–mesenchymal transition markers which, combined, can be used for the diagnosis and monitoring of therapy.

## 1. Introduction

Breast cancer (BC) is the type of cancer with the highest incidence and mortality rate in women in the world [1]; solid tumors metastasis is the major cause of cancer-related death, responsible for about 90% of breast cancer deaths [2,3]. Markers for the early detection of metastasis in all stages of BC [4], as well as a better understanding of the mechanism of its formation, may contribute to improving survival rates. 

The literature suggests that the metastatic cascade is composed of the following steps: (a) tumor cells separating from the primary tumor after changes in phenotype; (b) penetrating the basement membrane and invading neighboring tissues; (c) diffusing into the blood and/or lymphatic vessels; (d) forming micrometastasis in distant organs; and (e) adapting and reprograming the surrounding stroma to develop macrometastasis [5,6]. Thus, tumor epithelial cells need to acquire the characteristics to become circulating tumor cells (CTCs) and form metastasis. Such abilities are provided by a change in phenotype, such that cells tend to lose epithelial characteristics and start expressing mesenchymal characteristics [7,8,9]. This process is called epithelial–mesenchymal transition (EMT), but the mechanism of CTC formation has not yet been fully clarified. 

During the EMT process, tumor cells lose their expression of specific epithelial markers, including epithelial cadherin (E-cadherin), epithelial cell adhesion molecule (EpCAM), and cytokeratins (CKs), and acquire the expression of cytoskeletal mesenchymal markers, adhesion proteins, and stem-cell-like proteins such as vimentin, neural cadherin (N-cadherin), cluster of differentiation-44 (CD44), and aldehyde dehydrogenase 1 (ALDH1). In addition, they positively regulate metalloproteinases (MMP2 and MMP9) [10,11]. Consequently, CTCs progress from tumor epithelial cells to tumor stem cells (CSCs) through the EMT process. These stem cell characteristics not only enable migration and invasion by these cells, but also provide resistance to conventional therapies [12,13,14].

These markers are widely used in breast cancer. However, their expression level may be different in CTCs compared with tumor tissue. For example, one study has shown that there is a decrease in ADH/ALDH activity in breast tumor tissue compared with normal parenchyma [15]. On the other hand, the same authors, in another study, found that in the serum of patients with stage IV breast cancer, there is an increase in ADH isoenzyme 1 [16]. Despite these controversial findings in the ADH/ALDH pathway, there is a consensus regarding the use of ALDH1 as a stem cell marker commonly associated with epithelial–mesenchymal transition [17,18,19,20].

CTCs were thus identified as responsible for the dissemination of tumors to other organs of the body; therefore, the development of sensitive and specific assays for its detection the focus of contemporary translational research [21]. Despite recent advances, only one technology for the detection of CTCs (CellSearch^®^) has been approved by the Food and Drug Administration (FDA) for use in routine clinical practice [22,23]. In addition, it has an important limitation because it uses EpCAM as a marker for positive selection in the enrichment phase. However, CTCs show several phenotypes as a result of EMT, which implies that mesenchymal CTCs cannot be captured by this platform [24].

In this sense, we aimed to develop a panel of biomarkers for the diagnosis and treatment monitoring of breast cancer, through the detection of epithelial and mesenchymal markers in CTCs, purposing a new method of liquid biopsy without preview enrichment that could be a new effective tool for clinical routine. 

## 2. Results

### 2.1. Characteristics of Patients

The study included, in the first phase, 87 patients: 25 women diagnosed with benign breast disease (BBD) and 62 with BC. The mean age of the BBD group was 50 ± 14.8 years, and 54.76 ± 11 years for the BC group. The most frequent types of BBD were fibrocystic breast changes (n = 12), ductal hyperplasia (n = 5), fibroadenoma (n = 5), duct ectasia (n = 4), and fat necrosis (n = 4). The same patient may have had more than one type (Table S1). At the second phase of data collection, 23 patients were included in the study—6 control women, 6 women diagnosed with BBD, and 11 BC patients with 6 of them having not yet started CT (BC without CT), and 5 BC patients having concluded CT (BC with CT). The most frequent types of BBD were fibrocystic breast changes (n = 2) and benign papillary lesions (n = 2). The same patient may have had more than one BBD type (Table S1). The clinical, hormonal, and therapeutic data of the BC group are shown in Table 1. At the first phase, there were 9 patients out of 62 (14.5%) with a tumor in situ, 16 patients (25.8%) with T1 tumors, 24 patients (38.7%) with T2 tumors, and 5 patients (8.1%) with T3/T4 tumors. In total, 31 patients (50%) presented negative lymph nodes for the disease (N0), 14 (22.6%) had one to three positive lymph nodes (N1), and 7 (11.3%) presented more than three positive lymph nodes with tumor cells (N2/N3). Out of the 62 patients, 10 (16.1%) presented tumor stage 0; 10 patients also presented tumor stage IA; 8 (12.9%) presented tumor stage IIB; 7 (11.3%) presented tumor stage IIIA; and 2 (3.2%) presented tumor stage IIIB. Table S2 details the tumor staging of the studied groups (BC with chemotherapy and BC without chemotherapy), and is available in the Supplementary Material. Out of the 62 patients, 7 (27.4%) had histological grade 1 (G1) tumors, 27 (43.5%) presented histological grade 2 (G2), and 16 (25.8%) presented histological grade 3 (G3). Regarding the molecular subtype, 8 patients (12.9%) had estrogen receptor (ER)-negative, progesterone receptor (PR)-negative, human epidermal growth factor receptor 2 (HER2)-negative, and CK5/6-positive or epidermal growth factor receptor (EGFR)-positive; 8 patients had ER-, PR-, and HER2+; 21 patients (33.9%) had ER+ and/or PR+, HER2-, and Ki67 < 14%; 7 patients (11.3%) had ER+ and/or PR+, HER2-, and Ki67 ≥ 14; and 16 (25.8%) had ER+ and/or PR+ and HER2+. Most of the patients—49 (79%)—had not yet started CT (BC without CT); 13 BC patients had concluded CT (BC with CT).

In the second phase, there were 7 patients (63.6%) with T2 tumors, and 3 patients (27.3%) with T3 tumors. There were 5 patients (45.4%) who presented lymph nodes negative for disease (N0), 3 patients (27.3%) who had one to three positive lymph nodes (N1), and 1 (9.1%) presenting more than three lymph nodes positive for the presence of tumors cells (N2/N3). Out of 11 patients, 3 (27.3%) had histological grade 2 tumors and 7 (63.6%) had histological grade 3. Two patients (18.2%) had ER-, PR-, HER2-, and CK5/6+ or ERGF+. One patient (9.1%) had ER-, PR-, and HER2+, and one patient had ER+ and/or PR+, HER2-, and Ki67 < 14. Three patients (18.2%) had ER+ and/or PR+ and HER2+.

### 2.2. Transcriptional Levels of Target Markers

In Table 2, we present the comparisons between BC and BBD for the transcriptional levels of the target genes. No markers presented a statistically significant difference.

When the BC group was subdivided into women who underwent chemotherapy (BC with CT) and those who did not receive this treatment (BC without CT), there was a significant difference in peripheral blood for epithelial 2 expression. In group BC without CT, the median of the transcriptional level of epithelial 2 was higher compared with the BC with CT group, as well as the BBD group compared with the BC with CT group. The markers epithelial 4, mesenchymal 4, and mesenchymal 6 showed significantly higher levels in their transcripts in the BC without CT group compared with the BBD group. Mesenchymal 2 showed higher transcriptional levels in the BC without CT group compared with the BC with CT group and the BBD group (Table 3). Regarding transcriptional levels in breast tissue, the markers did not present significant differences when comparing the groups.

### 2.3. Transcriptional Levels of Target Markers and Primary Tumor Characteristics

We found no significant differences in means between the markers and the clinical characteristics of the primary tumor in the breast tissue of BC patients, independent of CT. However, we identified a significant difference in the peripheral blood between mesenchymal 6 and pathological staging; this difference of means was found between in situ (stage 0) and IIA and IIB tumors.

**Figure 1:**
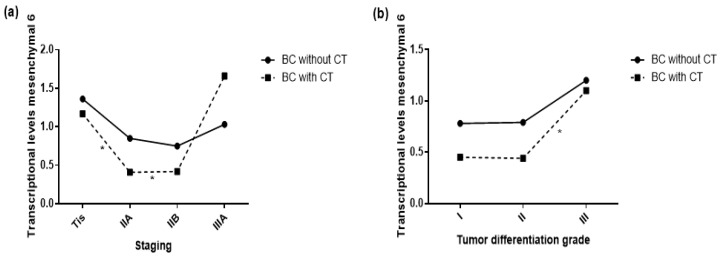
Comparison of means between mesenchymal marker 6 transcriptional levels and primary tumor characteristics: (**a**) comparison of means between mesenchymal marker 6 transcriptional levels and pathological staging; (**b**) comparison of means between mesenchymal marker 6 transcriptional levels and tumor differentiation grade. * *p* < 0.05. SD: (**a**) values shown in Table 4; (**b**) values shown in Table 5.

The transcriptional levels were higher in the in situ tumor, but exhibited a decrease in the intermediate stages and were then higher in later stages. This pattern had a tendency to repeat for the mesenchymal 2 and mesenchymal 4 markers (Table 4 and Figure 1a).

When gene transcriptional levels were correlated to tumor differentiation grade, we verified a similar pattern to that of pathological staging. There was an observed difference between histological grades 2 and 3 in the peripheral blood for mesenchymal 6, and the same tendency was also observed for decreased mesenchymal 2 levels in the intermediate grade, with a return to high levels at histological grade 3 (Table 5 and Figure 1b).

### 2.4. Populations of CTCs

For the protein analysis, with the results obtained from the qPCR and the literature data [25], the markers epithelial 6, mesenchymal 6, and mesenchymal 2 were selected. For the negative selection, the marker pan-leukocyte (CD45) was used. Using the presence or absence of these markers, three populations of CTC were defined: mesenchymal (CD45- epithelial 6- mesenchymal 6+ mesenchymal 2+), intermediate (CD45- epithelial 6+ mesenchymal 6+ mesenchymal 2+), and epithelial (CD45- epithelial 6+ mesenchymal 6- mesenchymal 2-). Before analyzing the populations of interest, the percentage of living cells was verified by means of propidium iodide labeling. On average, the percentage of living cells was 91.61%, with none of the samples presenting less than 80%.

**Figure 2:**
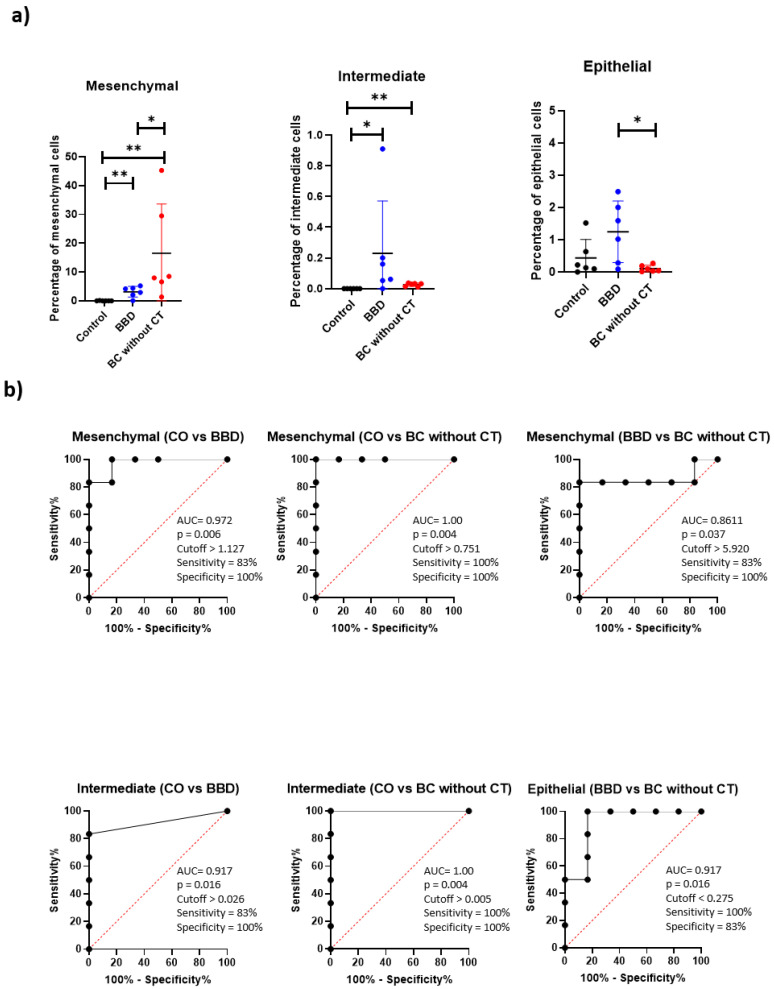
Diagnostic panel. (**a**) Scatter plot of percentage of mesenchymal CTCs (CD45- epithelial 6- mesenchymal 6+ mesenchymal 2+), intermediate CTCs (CD45- epithelial 6+ mesenchymal 6+ mesenchymal 2+), and epithelial CTCs (CD45- epithelial 6+ mesenchymal 6- mesenchymal 2-) for the control group (black), benign breast disease (blue), and breast cancer without treatment (red). The line represents the median, and the error bars (whiskers) represent the percentiles (* *p* < 0.05, ** *p* < 0.005, comparison of groups via the Mann–Whitney test). (**b**) ROC curves made from the percentage mesenchymal CTCs, intermediate CTCs, and epithelial CTCs for control vs. BBD, control vs. BC without CT, and breast benign disease vs. BC without CT. Results of the area under the curve (AUC), *p* value, cutoff, sensitivity, and specificity are shown near the ROC curve.

In the analyzed groups, there was a significant difference for the mesenchymal population, with a significantly lower percentage of these cells in the control group compared with the BC without CT and BBD group (*p* = 0.0043 and *p* = 0.0022, respectively). Additionally, the BBD group presented a significantly lower percentage of mesenchymal CTCs (*p* = 0.0041). In the intermediate profile, the control group presented a significantly lower percentage compared with the BBD and the BC without CT (*p* = 0.0152 and *p* = 0.0022, respectively). There was a significant difference for the epithelial CTCs in the BBD compared with the BC without CT group (*p* = 0.0152) with a high percentage of these CTCs in the BBD group (Figure 2a). These findings formed the diagnosis panel. 

A significantly higher percentage of CTC with the intermediate profile was found in the BC without CT group compared with the BC with CT group (*p* = 0.0043). The opposite occurred with CTCs with an epithelial profile (*p* = 0.0043). These findings formed the panel for the treatment monitoring. 

Through the analysis of CTC by flow cytometry, without previous enriching of these cells, it was possible to identify well-defined populations, enabling the optimization and simplification of the methodology, indicating its utility in clinical practice for diagnostics and treatment monitoring of breast cancer. 

### 2.5. Pre-Validation of Diagnostic Panel by ROC Curve

The ROC curve analysis showed good accuracy of the mesenchymal population of CTCs to discriminate the control women from the BBD and the BC without CT, with an AUC of 0.972 for control patients vs. BBD and an AUC of 1.00 for control vs. BC without CT. Using the ROC curve, it was possible to select the optimal cutoff that distinguished the control women from the BBD and BC. This yielded a sensitivity of 83% and a specificity of 100% for control vs. BBD, and a sensitivity and a specificity of 100% for control vs. BC without CT. The ROC curve analysis showed a reasonable accuracy of the mesenchymal population of CTCs to discriminate BBD patients from BC without CT, with an AUC of 0.861, a sensitivity of 83%, and a specificity of 100%. The intermediate population of CTCs discriminated the control women from the BBD and BC without CT, with an AUC of 0.917 for control patients vs. BBD and an AUC of 1.00 for control vs. BC without CT. Control vs. BBD showed a sensitivity of 83% and a specificity of 100%, and control vs. BC without CT showed a sensitivity and a specificity of 100%. The epithelial population of CTCs discriminated benign patients from BC without CT with an AUC of 0.917, a sensitivity of 100%, and a specificity of 83% (Figure 2b). 

The ROC curve revealed that the populations of CTCs exhibited excellent diagnostic efficiency for distinguishing between women with breast cancer, women with benign disease and women without alterations (control).

### 2.6. Pre-Validation of Treatment Monitoring Panel by ROC Curve 

**Figure 3:**
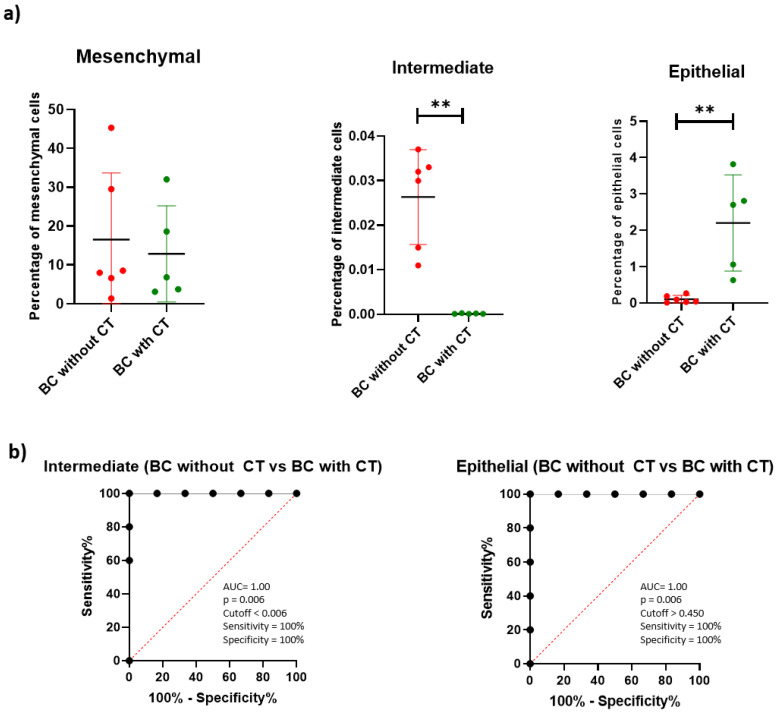
Panel for treatment monitoring. (**a**) Scatter plot of percentage of mesenchymal CTCs (CD45- epithelial 6- mesenchymal 6+ mesenchymal 2+), intermediate CTCs (CD45- epithelial 6+ mesenchymal 6+ mesenchymal 2+), and epithelial CTCs (CD45- epithelial 6+ mesenchymal 6- mesenchymal 2-) for breast cancer without treatment (red) and breast cancer with treatment (green). The line represents the median, and the error bars (whiskers) represent the percentiles (** *p* < 0.005, comparison of groups via the Mann–Whitney test). (**b**) ROC curves made from the percentage of intermediate CTCs and epithelial CTCs for BC without CT vs. BC with CT. Results about area under the curve (AUC), *p* value, cutoff, sensitivity, and specificity are being shown near the ROC curve.

The ROC curve analysis showed good accuracy of the intermediate and epithelial population of CTCs to discriminate the BC without CT from the BC with CT group, with both presenting an AUC of 1.00, a sensitivity of 100%, and a specificity of 100% (Figure 3b). 

These results show that it may be possible to use this panel for monitoring the treatment of patients with breast cancer. 

## 3. Discussion

In our study, we distinguished specific and well-defined CTC populations by flow cytometry, without the need for prior enriching. First, the analysis of transcriptional levels of EMT markers in blood and tissue made it possible to select three markers to compose the detection panel together with CD45 for the negative selection of CTCs. To capture different phenotypes of CTCs, we analyzed different combinations of mesenchymal and epithelial markers by flow cytometry, obtaining a diagnostic panel with optimal accuracy to not only differentiate women without breast alterations, but also benign breast disease from breast cancer.

Transcriptional levels of mesenchymal 2 were elevated in the BC without chemotherapy group compared with the BC with chemotherapy group and BBD. This is a classic biomarker of the mesenchymal phenotype [25,26,27]. Studies have shown that higher levels of this protein are associated with a greater invasion power of tumor cells in vitro and, consequently, mesenchymal 2 silencing has been shown to reduce the formation of metastasis [28,29,30]. The mesenchymal 6, another established marker of stem cells, had higher transcript levels in BC without chemotherapy compared with the BBD group and significant differences when compared with the mean of mesenchymal 6 transcriptional levels and primary tumor characteristics. Characterized as an adhesion molecule, it is multifunctional and multistructural. It belongs to the family of transmembrane glycoproteins, related to cell-to-cell and cell–matrix interactions [31,32]. Through these interactions, mesenchymal 6 promotes the invasion and migration of CTCs [33,34]. We selected these markers because these results and characteristics are very important to identifying mesenchymal phenotypes. 

Although the epithelial 6 marker did not significantly differentiate the groups in our transcriptional analyses, we selected this marker because of the technologies developed thus far for the detection of CTCs, it is the most commonly used [35,36]. This is understandable because epithelial 6 is a transmembrane glycoprotein involved not only in cell-to-cell adhesion, but also in the regulation of proliferation, migration, stemness, and EMT in tumor cells [37].

Based on the analyses of expression of mesenchymal, epithelial, and CD45, in our study, combining these markers, we determined three CTCs phenotypes: mesenchymal, intermediate, and epithelial. We observed various CTCs phenotypes in each patient, but not in the control group. This result was confirmed by data regarding the presence of intravasation mechanisms of tumor cells with various initial phenotypes [38,39,40].

In our study, the population of mesenchymal CTCs presented significantly higher percentages in the BC group, corroborating previous studies where the number of mesenchymal CTCs was significantly higher than the epithelial-positive [41]. In this way, our method eliminates an important limitation experienced by most CTC detection platforms developed to date [42]. Furthermore, we were able to differentiate BBD from BC, which would provide a less invasive tool for the diagnosis of these benign changes, which, to date, are differentiated from BC only by tissue biopsy [43,44].

Several studies have reported that an intermediate profile could confer greater aggressiveness and invasiveness on CTCs [45,46]; however, there are higher percentages of these cells in both the BBD and BC groups compared with the control group. Epithelial CTCs, on the other hand, showed higher percentages in the control and BBD groups, differentiating the BBD from the BC group, which can be associated with the detection of the other types of cells, such as circulating endothelial cells [47]. However, this fact did not limit our detection method, which included the analysis of other CTC profiles.

Tumor diagnosis includes several steps such as image analysis, tissue biopsy, and blood and genetic tests [48]. None of these techniques alone is sufficient to obtain the diagnosis, and they can be highly invasive. For example, mammography, the gold standard for screening, presents controversial data on efficacy and cost-effectiveness [49]. Our panel of biomarkers for the diagnosis of breast cancer was composed of the three subpopulations of CTCs. It had a sensitivity and specificity between 80% and 100% and did not have most of the limitations of the methods used in routine clinical practice. However, it needs to be validated in a larger population and associated with clinical and primary tumor characteristics.

The BC without chemotherapy group showed a different pattern of intermediate and epithelial CTCs from the group with BC with chemotherapy, with higher percentages of intermediate CTCs in the BC without CT and higher percentages of epithelial CTCs in the BC with CT. This treatment monitoring panel presented a specificity and sensibility of 100% to identify BC with CT. A stage III clinical trial with 319 patients also evaluated changes in CTCs and found that patients who had persistently elevated levels of CTCs had poor overall survival, confirming the impact of CTC analysis on monitoring and decision-making in the treatment of BC [50]. Prospective studies are needed to confirm whether these changes in the profile of CTCs in our panel of biomarkers is indicative or not of chemotherapy efficacy and whether they could guide treatment decision-making. 

In summary, we show that transcriptional levels of some EMT markers change significantly in BBD, BC without CT, and BC with CT in blood, but not in tissue. CTC analyses presented three phenotypes of CTCs with different expression patterns for each group. Therefore, these biomarkers combined could be used for the diagnosis and monitoring of treatment with high sensitivity and specificity.

## 4. Materials and Methods

### 4.1. Studied Patients 

This experimental study was conducted at the Federal University of Uberlandia (UFU) and included interviews with patients diagnosed with BC and BBD as well as women who had bilateral Breast Image Reporting and Data System 1 (BIRADS 1) as a result of mammography (the control group). Data were collected at two phases from different patients, and they were invited to participate while waiting for surgery in the hospital waiting room. Firstly, the transcriptional profiles were evaluated from 2013 to 2016 and, secondly, the protein profiles were analyzed in 2019. 

Women who presented a BC diagnosis, confirmed by anatomopathological examination, were part of the BC group. Women who, after surgery, did not present BC but presented fibroadenoma, atypical ductal hyperplasia, papilloma or other benign breast diseases constituted the BBD group. Women who had bilateral BIRADS 1 as a result of mammography formed the control group. Women were evaluated regardless of previous neoadjuvant chemotherapy or not. In this study, patients under the age of 18 years, with primary tumors in locations other than the breast and those who were mentally or physically unable to respond to the interviews, were excluded. 

This study was approved by the Human Research Ethics Committee (protocol number 174.009/2013), and the entire study was conducted based on the Helsinki Declaration standards. All participants signed a consent form. 

### 4.2. Analyses of Transcriptional Levels

#### 4.2.1. Sample Collection 

Peripheral blood and mammary tumor tissue samples were obtained for subsequent analysis of the expression of specific genes. Peripheral blood samples were collected in a VacutainerTM tube containing 7.2 mg of K2-EDTA. The obtained tissue was stored at −80 °C submerged in RNAlater (Invitrogen™) for ribonucleic acid (RNA) extraction.

#### 4.2.2. Extraction of Total RNA from Peripheral Blood and Tissue

RNA was extracted using Trizol (Invitrogen, Life Technologies, Carlsbad, CA, USA), following the manufacturer’s recommendations. The extraction product was subjected to agarose gel electrophoresis (1.5% agarose and 0.5 μg/mL ethidium bromide) made in TBE buffer (45 mM Tris-borate, pH 8.3 and 1 mM EDTA). After 1 h at 100 V, the electrophoretic profile was visualized under UV light and documented using the VDS ImageSystem (Amersham Biosciences) to evaluate the quality of RNA extraction.

#### 4.2.3. Reverse Transcription

For reverse transcription, 1 μg of total RNA, 10 U of RNase inhibitor (Invitrogen), 40 U of MMLV-RT (Amersham Biosciences), 1X of MMLV-RT buffer (Amersham Biosciences), 200 μM dNTPs (Invitrogen), and 126 pmoles of primer (Invitrogen) were used. The solution was incubated in a PTC-100 thermocycler (MJ Research) at 37 °C for 1 h and heated at 95 °C for 5 min [51]. Control reactions (without RNA) were performed to verify possible exogenous contaminants. The complementary deoxyribonucleic acid (cDNA) was stored at −20 °C for further amplification.

#### 4.2.4. PCR for Sample Validation

The quality of the cDNA was assessed by the amplification of a 536 bp fragment of the constitutive gene β2-microglobulin (B2M) flanked by the oligonucleotides: 5′-AGCAGAGAATGGAAAGTCAAA 3′ and 5′ TGTTGATGTTGGAGAGAGAA 3′. For this reaction, 1X PCR buffer (20 mM Tris-HCl—pH 8.0, 0.1 mM EDTA, 1 mM DTT, 50% *v*/*v* glycerol) (Invitrogen), 200 μM dNTPs (Invitrogen), 5 pmoles of primer (Invitrogen), 1 U Platinum^®^Taq DNA polymerase (Invitrogen^®^), and 4 mM MgCl2 (Invitrogen^®^) were used. Each reaction comprised the following steps: the sample was heated to 95 °C for 4 min; then, cycles of 94 °C for 40 s, 59 °C for 40 s, 72 °C for 50 s were repeated 34 times and finally left for 5 min at 72 °C in a PTC-100 thermal cycler (MJ Research Inc.). The product of the reaction was analyzed by agarose gel electrophoresis.

#### 4.2.5. Analysis of Specific Oligonucleotides

In order to establish the transcriptional profile of mesenchymal and epithelial markers in patients with BC and BBD, pairs of oligonucleotides were designed for each of the gene sequences and for the reference gene B2M. Oligonucleotide flanking fragments in sizes of 50 to 150 base pairs and that were considered viable for amplification standards according to Primer Express version 3.0 software (Applied Biosystems) were used.

#### 4.2.6. Relative Transcriptional Quantification by qPCR

The relative transcriptional quantifications of the target genes were estimated by means of real-time PCR (qPCR) from the obtained cDNA. Samples were amplified in duplicate, and the detection occurred from the fluorescence emission of SYBR^®^Green dye in accordance with Master Mix SYBR^®^Green PCR Core Reagents kit (Applied Biosystems). 

### 4.3. CTC Characterization by Flow Cytometry

Analysis of the proteins involved in EMT in the CTCs was performed by flow cytometry using the BD ACCURI^TM^ C6 (Becton, Dickinson and Company (BD), Franklin Lakes, FL, USA). Steps, previously to flow cytometry, of the isolation and/or enrichment of CTCs, were not carried out. For this experiment, two tubes containing peripheral blood stabilized with EDTA were used. The first tube was discarded to avoid contamination with epithelial cells. The sample was first submitted to centrifugation, and then the leukocyte monolayer was collected. The leukocyte monolayer was incubated with AB serum to block the portion FC and then with fluorochrome-labeled monoclonal antibodies to CD45 (304008, PE) (Biolegend, San Diego, CA, USA), mesenchymal 6 (PE/Cy7) (Biolegend, San Diego, CA, USA), and epithelial 6 (APC) (Biolegend, San Diego, CA, USA). Then, erythrocytes were lysed in lysis solution (BD FACS lysing solution) and washed twice with wash solution (Phosphate-Buffered Saline 1x/Bovine Serum Albumin 1%/Sodium azide 0.1%). The cell pellet was resuspended in 150 μL of wash solution for extracellular markings. For intracellular markings, the cell pellet was incubated with a permeabilizing solution (BD FACS permeabilizing solution 2). Then, the sample was incubated with AB serum, washed twice with wash solution and then with fluorochrome-labeled monoclonal antibody to mesenchymal 2 (Alexa fluor 488) (Becton, Dickinson, and Company (BD), Franklin Lakes, FL, USA) and washed with wash solution. The cell pellet was resuspended in 150 μL of wash solution. 

**Figure 4:**
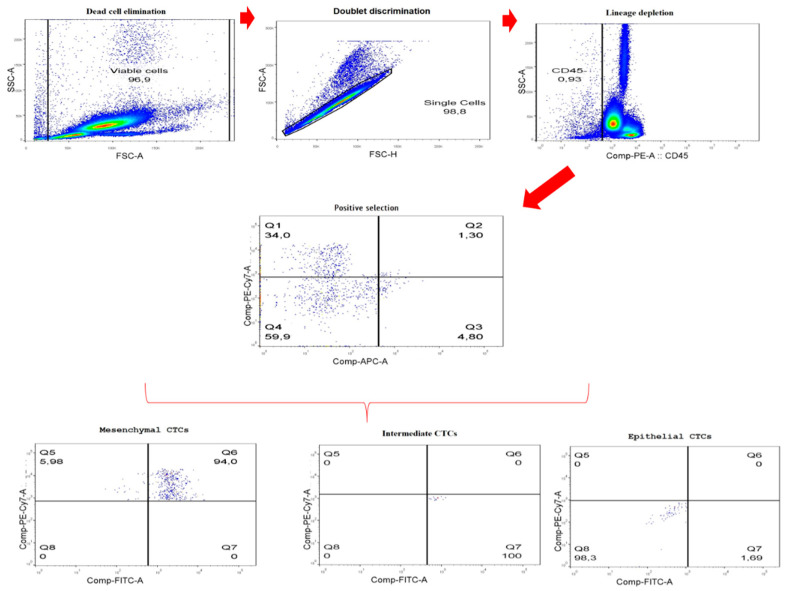
Representative scheme of the gating strategy using the CMP02 sample.

Measurements were performed against unstained cells, compensation beads, propidium iodide (to measure the percentage of viable cells), and isotype control antibodies to PE (400112) (Biolegend, San Diego, CA, USA), PE/Cy7 (400126) (Biolegend, San Diego, CA, USA), APC (400111) (Biolegend, San Diego, CA, USA), and Alexa fluor 488 (557721) (Becton, Dickinson and Company (BD), Franklin Lakes, FL, USA). On average, 300,000 events were collected every sample. A particular order was followed to analyze the data: (i) cell debris, (ii) doublet elimination, (iii) elimination of total leukocytes marked with pan-leukocyte marker (antibody CD45), and (iv) positive selection of mesenchymal (CD45- epithelial 6- mesenchymal 6+ mesenchymal 2+), intermediate (CD45- epithelial 6+ mesenchymal 6+ mesenchymal 2+), or epithelial (CD45- epithelial 6+ mesenchymal 6- mesenchymal 2-) CTCs (Figure 4). Data were analyzed using FlowJo software (version 10.0.7; Tree Star, Ashland, OR, USA) with percentage CTC populations in relation to the population of CD45-negative.

### 4.4. Statistical Analysis

Initially, the normality test was performed. From the behavior of the variables, parametric tests were performed for variables with normal distribution, or non-parametric tests for variables without normal distribution. The specific tests applied are indicated in the legends of the figures. The 95% confidence interval was considered and *p* <0.05 values were considered significant. Statistical analyses were performed on GraphPad Prism 5 (GraphPad Software, La Jolla, CA, USA) and SPSS version 21.0 (SPSS, IBM, Chicago, IL, USA). 

## 5. Patents

There is a patent resulting from the work reported in this manuscript submitted to the National Institute of Industrial Property of Brazil, process number BR 10 2020 026395 1 and PCT request number PCT/BR2021/050561. 

## Figures and Tables

**Table 1:** Clinical, hormonal, and therapeutic characteristics of breast cancer patients.

	1º Phase		2º Phase	
Variable	Patients (n = 62)		Patients (n = 11)	
	n	%	n	%
Age (years)				
Mean ± SD	54.76 ± 11.00		53.80 ± 9.13	
Tumor size				
pTis	9	14.5	0	0.0
pT1	16	25.8	0	0.0
pT2	24	38.7	7	63.6
pT3	4	6.5	3	27.3
pT4	1	1.6	0	0.0
NR	8	12.9	1	9.1
Regional Lymph nodes				
pN0	31	50.0	5	45.4
pN1	14	22.6	3	27.3
pN2	5	8.1	1	9.1
pN3	2	3.2	0	0.0
NR	10	16.1	2	18.2
Tumor Stage				
0	10	16.1	0	0
IA	10	16.1	0	0
IIA	15	24.2	5	45.4
IIB	8	12.9	4	36.4
IIIA	7	11.3	1	9.1
IIIC	2	3.2	0	0.0
NR	10	16.1	1	9.1
Histological grade				
G1	7	11.3	0	0.0
G2	27	43.5	3	27.3
G3	16	25.8	7	63.6
NR	12	19.4	1	9.1
Molecular subtyp				
ER-, PR-, HER2-, and CK5/6+ or EGFR+	8	12.9	2	18.2
ER-, PR-, and HER2+	8	12.9	1	9.1
ER+ and/or PR+, HER2-, and Ki67 < 14	21	33.9	1	9.1
ER+ and/or PR+, HER2-, and Ki67 ≥ 14	7	11.3	3	27.2
ER+ and/or PR+ and HER2+	16	25.8	2	18.2
NR	2	3.2	2	18.2
Chemotherapy				
No	49	79.0	6	54.5
Yes	13	21.0	5	45.5

**Table 2:** Median/mean and percentiles/SD of transcriptional levels of molecular markers.

Markers		BC		BBD	*p* Value
	n	Median (p25-p75)/Mean(±SD)	n	Median (p25-p75)/Mean(±SD)	
Bloodstream					
Epithelial 1 ^1^	36	0.45 (0.16–0.90)	15	0.60 (0.44–1.77)	0.097
Epithelial 2 ^1^	36	0.60 (0.27–1.49)	15	0.96 (0.24–1.84)	0.656
Epithelial 3 ^1^	36	1.11 (0.49–2.27)	16	0.74 (0.59–1.56)	0.868
Epithelial 4 ^1^	35	1.71 (0.84–3.43)	15	0.82 (0.38–1.82)	0.113
Epithelial 5 ^1^	29	1.68 (0.83–3.46)	12	1.04 (0.78–2.25)	0.271
Epithelial 6 ^1^	28	1.39 (0.35–3.22)	13	1.31 (0.43–5.14)	0.746
Mesenchymal 1 ^2^	17	0.84 (0.95)	8	1.09 (1.07)	0.574
Mesenchymal 2 ^1^	24	2.07 (0.86–4.11)	9	0.92 (0.45–1.99)	0.154
Mesenchymal 3 ^1^	28	1.69 (0.70–3.69)	12	1.08 (0.73–2.45)	0.590
Mesenchymal 4 ^1^	27	1.75 (0.66–4.35)	12	0.74 (0.38–2.47)	0.086
Mesenchymal 5 ^1^	29	0.78 (0.47–1.17)	13	0.64 (0.18–1.06)	0.174
Mesenchymal 6 ^2^	29	0.78 (0.51)	12	0.54 (0.38)	0.148
Tissue					
Epithelial 5 ^1^	25	0.56 (0.20–1.14)	11	0.83 (0.44–2.85)	0.359
Epithelial 6 ^1^	20	0.63 (0.36–1.57)	11	1.46 (0.35–2.08)	0.427
Mesenchymal 1 ^1^	28	0.96 (0.17–58.42)	13	1.22 (0.23–3.34)	0.628
Mesenchymal 2 ^1^	16	0.66 (0.54–2.66)	10	1.24 (0.24–2.66)	0.897
Mesenchymal 4 ^1^	18	0.91 (0.35–8.38)	11	0.45 (0.23–3.66)	0.550
Mesenchymal 5 ^1^	19	0.56 (0.05–6.66)	10	2.17 (0.12–6.54)	0.573

^1^ Mann–Whitney test. ^2^ Independent samples *t*-test.

**Table 3:** Influence of chemotherapy on transcriptional levels of target genes.

Markers		BC without CT		BC with CT		BBD
	n	Median (p25-p75)/Mean(±SD)	n	Median (p25-p75)/Mean(±SD)	n	Median (p25-p75)/Mean(±SD)
Bloodstream						
Epithelial 1 ^1^	15	0.57 (0.23–1.02)	21	0.33 (0.12–0.84)	15	0.60 (0.44–1.77)
Epithelial 2 ^1^	15	1.05 (0.46–1.97) ^a^	21	0.59 (0.13–0.65) ^b^	15	0.96 (0.24–1.84) ^a^
Epithelial 3 ^1^	15	1.63 (0.61–2.30)	21	0.83 (0.45–2.10)	16	0.74 (0.59–1.56)
Epithelial 4 ^1^	15	2.21 (0.95–2.77) ^a^	20	1.59 (0.65–3.67) ^a,b^	15	0.82 (0.38–1.82) ^b^
Epithelial 5 ^1^	13	1.78 (0.68–4.91)	16	1.64 (0.97–2.18)	12	1.04 (0.78–2.25)
Epithelial 6 ^1^	12	1.80 (0.80–2.76)	16	0.89 (0.15–5.20)	13	1.31 (0.43–5.14)
Mesenchymal 1 ^2^	10	0.94 (1.20)	7	0.70 (0.46)	8	1.09 (1.07)
Mesenchymal 2 ^1^	13	2.65 (1.79–6.09) ^a^	11	1.27 (0.53–2.05) ^b^	9	0.92 (0.45–1.99) ^b^
Mesenchymal 3 ^1^	13	1.79 (0.87–5.54)	15	1.61 (0.58–2.41)	12	1.08 (0.73–2.45)
Mesenchymal 4 ^1^	12	2.16 (0.88–5.72) ^a^	15	1.24 (0.64–2.58) ^a,b^	12	0.74 (0.38–2.47) ^b^
Mesenchymal 5 ^1^	13	0.83 (0.53–1.25)	16	0.70 (0.37–1.17)	13	0.64 (0.18–1.06)
Mesenchymal 6 ^2^	13	1.04 (0.49) ^a^	16	0.58 (0.44) ^a,b^	12	0.54 (0.38) ^b^
Tissue		^a^				
Epithelial 5 ^1^	20	0.48 (0.20–0.88)	5	0.78 (0.20–23.4)	11	0.83 (0.44–2.85)
Epithelial 6 ^1^	15	0.64 (0.34–1.66)	5	0.50 (0.32–4.6)	11	1.46 (0.35–2.08)
Mesenchymal 1 ^1^	21	0.59 (0.15–66.85)	7	9.33 (0.15–62.03)	13	1.22 (0.23–3.34)
Mesenchymal 2 ^1^	12	0.66 (0.53–4.90)	4	1.55 (0.56–2.66)	10	1.24 (0.24–2.66)
Mesenchymal 4 ^1^	15	2.75 (0.36–10.09)	3	0.45 (0.014–NA)	11	0.45 (0.23–3.66)
Mesenchymal 5 ^1^	19	0.74 (0.05–7.69)	4	0.38 (0.06–0.92)	10	2.17 (0.12–6.54)

^1^ Mann–Whitney test. ^2^ Independent samples *t*-test and Tukey post hoc test at the 5% probability level. NA: not available due to the small sample size.

**Table 4:** Comparison of means between transcriptional levels of target genes and pathological staging.

Markers	n	0 Median (p25-p75) Mean (±SD)	n	IA Median (p25-p75) Mean (±SD)	n	IIA Median (p25-p75) Mean (±SD)	n	IIB Median (p25-p75) Mean (±SD)	n	IIIA Median (p25-p75) Mean (±SD)	*p* Value
Bloodstream											
Epithelial 1 ^1^	6	0.86 (0.28–5.47)	5	0.33 (0.15–0.71)	7	0.77 (0.04–2.28)	6	0.42 (0.14–0.76)	4	0.63 (0.16–1.46)	0.77
Epithelial 2 ^1^	6	1.28 (0.44–4.39)	5	0.90 (0.15–1.74)	7	0.59 (0.01–2.06)	6	0.82 (0.14–2.74)	4	1.08 (0.66–1.45)	0.66
Epithelial 3 ^1^	6	1.61 (0.59–4.39)	5	1.88 (0.24–3.29)	7	0.51 (0.03–3.14)	6	1.24 (0.44–3.16)	4	1.86 (1.02–2.20)	0.56
Epithelial 4 ^1^	6	1.51 (0.58–7.56)	5	2.21 (0.38–4.37)	7	1.28 (0.06–2.39)	6	1.65 (0.97–4.57)	4	3.26 (1.98–7.76)	0.42
Epithelial 5 ^1^	5	1.28 (0.33–2.36)	2	1.24 (0.70–NA)	7	1.96 (0.03–3.50)	5	4.29 (1.28–7.09)	3	1.02 (0.10–NA)	0.44
Epithelial 6 ^1^	4	0.46 (0.05–1.40)	2	1.01 (0.02–NA)	7	0.50 (0.00–8.90)	5	1.67 (1.09–4.84)	3	2.86 (1.01–NA)	0.37
Mesenchymal 1 ^2^	4	1.14 (1.77)	1	0.69 (NA)	4	0.53 (0.42)	3	0.63 (0.50)	2	1.88 (0.47)	0.66
Mesenchymal 2 ^1^	4	2.01 (1.14–2.62)	2	1.11 (0.11–NA)	5	1.27 (0.04–2.57)	5	3.31 (1.39–10.20)	3	5.84 (0.53–NA)	0.27
Mesenchymal 3 ^1^	5	5.41 (0.26–11.59)	2	0.45 (0.20–NA)	7	1.05 (0.03–3.98)	4	2.91 (1.68–5.25)	3	1.39 (0.75–NA)	0.32
Mesenchymal 4 ^1^	4	1.16 (0.19–3.68)	2	5.82 (3.66–NA)	7	4.53 (0.81–6.18)	5	0.64 (0.43–1.69)	3	1.75 (0.58–NA)	0.11
Mesenchymal 5 ^1^	6	0.93 (0.69–3.23)	2	0.66 (0.55–NA)	8	1.05 (0.44–46.14)	5	0.49 (0.21–1.02)	3	1.43 (0.25–NA)	0.38
Mesenchymal 6 ^2^	6	1.32(0.63) ^a^	2	0.81 (0.03)	7	0.47 (0.30) ^b^	5	0.55 (0.19) ^b^	3	1.24 (0.40)	0.01
Tissue											
Epithelial 5 ^1^	4	0.53 (0.12–0.76)	4	0.28 (0.06–0.74)	6	4.92 (0.16–45.41)	5	0.64 (0.11–4.77)	3	1.39 (0.35–NA)	0.35
Epithelial 6 ^1^	4	0.48 (0.10–0.79)	3	0.86 (0.18–NA)	6	4.62 (0.03–8.40)	2	0.26 (0.01–NA)	2	1.06 (0.47–NA)	0.27
Mesenchymal 1 ^2^	4	21.92 (42.41)	6	76.65 (158.67)	8	100.16 (163.64)	4	45.30 (56.67)	3	45.63 (71.66)	0.84
Mesenchymal 2 ^1^	3	0.65 (0.65–NA)	3	0.86 (0.18–NA)	4	1.69 (0.52–10.34)	1	9.10 (NA)	2	1.31 (0.17–NA)	0.64
Mesenchymal 4 ^1^	3	0.93 (0.63–NA)	3	0.09 (0.01–NA)	4	13.41(0.46–45.95)	2	5.49 (0.89–NA)	3	0.45 (0.36–NA)	0.32
Mesenchymal 5 ^1^	3	6.65 (0.20–NA)	4	0.02 (0.02–1.25)	5	0.56(0.11–30.85)	3	16.75 (0.74–NA)	2	0.59 (0.15–NA)	0.17

^1^ Kruskal–Wallis test. ^2^ One-way ANOVA and Tukey post hoc test at the 5% probability level. NA: not available due to the small sample size.

**Table 5:** Comparison of means between transcriptional levels of the target genes and tumor differentiation grade.

Markers	n	1 Median (p25-p75) Mean (±SD)	n	2 Median (p25-p75) Mean (±SD)	n	3 Median (p25-p75) Mean (±SD)	*p* Value
Bloodstream							
Epithelial 1 ^1^	3	0.23 (0.17–NA)	16	0.51 (0.14–0.87)	7	0.33 (0.05–1.03)	0.848
Epithelial 2 ^1^	3	0.59 (0.17–NA)	16	0.60 (0.17–1.86)	7	0.60 (0.25–1.49)	0.823
Epithelial 3 ^1^	3	1.41 (0.45–NA)	16	0.73 (0.45–2.27)	7	1.90 (0.52–2.71)	0.633
Epithelial 4 ^1^	3	2.02 (1.01–NA)	16	1.63 (0.72–4.15)	7	2.76 (0.84–3.75)	0.745
Epithelial 5 ^1^	3	4.29 (0.10–NA)	14	1.99 (1.56–4.00)	5	0.95 (0.02–3.28)	0.226
Epithelial 6 ^1^	3	2.46 (0.01–NA)	14	1.82 (0.36–5.86)	5	1.15 (0.0 5–3.10)	0.645
Mesenchymal 1 ^2^	2	0.35 (0.17)	6	0.52 (0.42)	4	1.18 (0.86)	0.200
Mesenchymal 2 ^1^	3	7.86 (5.84–NA)	10	1.80 (0.15–2.76)	4	1.95 (0.72–5.41)	0.091
Mesenchymal 3 ^1^	2	3.53 (1.39–NA)	14	1.78 (0.62–2.21)	5	1.61 (0.40–3.52)	0.632
Mesenchymal 4 ^1^	3	1.16 (0.60–NA)	13	2.57 (1.63–5.19)	5	0.64 (0.31–4.83)	0.117
Mesenchymal 5 ^1^	3	1.04 (0.49–NA)	15	0.65 (0.35–1.03)	5	1.03 (0.21–24.07)	0.668
Mesenchymal 6 ^2^	3	0.67 (0.21) ª^,b^	15	0.56 (0.33) ^a^	4	1.12 (0.49) ^b^	0.032
Tissue							
Epithelial 5 ^1^	6	0.50 (0.15–33.60)	11	0.82 (0.21–1.39)	6	0.39 (0.29–6.21)	0.886
Epithelial 6 ^1^	4	0.68 (0.13–6.51)	8	0.66 (0.25–2.24)	6	0.54 (0.41–3.01)	0.993
Mesenchymal 1 ^2^	6	35.88 (48.86)	12	51.38 (95.64)	8	104.45 (181.85)	0.602
Mesenchymal 2 ^1^	3	2.73 (0.94–NA)	6	1.20 (0.53–4.43)	5	0.56 (0.10–0.68)	0.067
Mesenchymal 4 ^1^	3	10.09 (0.09–NA)	7	0.89 (0.45–4.34)	6	2.67 (0.26–17.34)	0.833
Mesenchymal 5 ^1^	4	0.65 (0.15–21.03)	8	0.69 (0.04–2.19)	5	0.15 (0.04–18.75)	0.928

^1^ Kruskal–Wallis test. ^2^ One-way ANOVA and Tukey post hoc test at the 5% probability. NA: not available due to the small sample size.

## Data Availability

Not applicable.

## Supplementary Materials

The following supporting information can be downloaded at: https://www.mdpi.com/article/10.3390/ijms231911983/s1.
