# Supplementary material for: A Novel Detection Method of Breast Cancer through a Simple Panel of Biomarkers
Source: Int J Mol Sci. 2022 Oct 9;23(19):11983. doi: 10.3390/ijms231911983 (PMC9570447; doi:10.3390/ijms231911983)
Supplement: Supplementary file 1 [file ijms-23-11983-s001.zip › ijms-1943685-supplementary.pdf]

Table S1. Characteristics of benign breast disease

ID	Diagnosis
**1ª Phase**	
M39	Fibrocystic breast changes, simple adenosis and ductal hyperplasia
M41	Atypical ductal hyperplasia and multifocal fat necrosis
M42	Benign lipochondromatous tumor of the breast
M51	Epithelial hyperplasia, sclerosing adenosis, fibrocystic breast changes, and apocrine metaplasia
M54	Fibrocystic breast changes and simple adenosis
M57	Encapsulated fat necrosis
M71	Extensive fat necrosis, sclerosing adenosis, columnar cell hyperplasia
M74	Breast hamartoma
M75	Encapsulated fat necrosis
M78	Atypical ductal hyperplasia focal
M79	Fibrocystic breast changes and ductal hyperplasia
M91	Fibrocystic breast changes, ductal ectasia and fibroadenoma
M105	Fibrocystic breast changes
M109	Fibrocystic breast changes, ductal hyperplasia and sclerosing adenosis
M113	Benign phyllodes tumor and ductal hyperplasia
M115	Columnar alterations with apical secretion, fibrocystic breast changes, and microcalcifications
M116	Fibroadenoma
M118	Fibrocystic breast changes and ductal hyperplasia
M171	Ductal ectasia and atypical ductal hyperplasia
M175	Ductal ectasia
M185	Fibroadenoma and fibrocystic breast changes
M187	Fibroadenoma
M198	Microcalcification, flat epithelial atypia, and fibrocystic breast changes
M202	Fibrocystic breast changes and ductal ectasia
M210	Giant Juvenile Fibroadenoma
**2ª Phase**	
CMP12	Fibrocystic breast changes and apocrine metaplasia
CMP20	Benign papillary lesions and chronic inflammation
CMP23	Benign papillary lesions, ductal ectasia, and fibrocystic breast changes
CMP24	Pericanalicular fibroadenoma associated with stromal hyperplasia
CMP25	Granulomatous mastitis
CMP28	Ductal hyperplasia and benign papillary lesions

Table S2. Frequency of tumor staging in BC without CT and BC with CT groups

	**1º Phase**				**2º Phase**			
	**BC without CT (n=49)**		**BC with CT (n=13)**		**BC without CT (n=6)**		**BC with CT (n=5)**	
**Tumor Stage**	**n**	**%**	**n**	**%**	**n**	**%**	**n**	**%**
**0**	9	18.4	1	7.7	0	0.0	0	0.0
**IA**	6	12.2	4	30.8	0	0.0	0	0.0
**IB**	0	0.0	0	0.0	0	0.0	0	0.0
**IIA**	12	24.5	3	23.1	3	50.0	2	40.0
**IIB**	7	14.3	1	7.7	3	50.0	1	20.0
**IIIA**	5	10.2	2	15.4	0	0.0	1	20.0
**IIIB**	0	0.0	0	0.0	0	0.0	0	0.0
**IIIC**	2	4.10	0	0.0	0	0.0	0	0.0
**NR**	8	16.3	2	15.4	0	0.0	1	20.0
